# Objective Biomarker Development for Parameter Optimization in Neuromodulation Using High-Density EMG Temporal and Spatial Features

**DOI:** 10.3390/bioengineering13070766

**Published:** 2026-06-30

**Authors:** Shirin Madarshahian, Nikoo Javadpour, Michael Trakhtorchuck, Tatiana Guerrero-David, Kristin Gustafson, James S. Harrop, Caio M. Matias, M. J. Mulcahey, Alessandro Napoli, Alexander Vaccaro, Mijail Serruya

**Affiliations:** 1 Department of Biostatistics and Health Data Science, College of Health, Lehigh University, Bethlehem, PA 18015, USA; 2 Good Shepherd Rehabilitation Network, Allentown, PA 18104, USA; 3 Department of Mechanical Engineering, P. C. Rossin College of Engineering & Applied Science, Lehigh University, Bethlehem, PA 18015, USA; nij424@lehigh.edu; 4 Raphael Center for Neurorestoration, Thomas Jefferson University Hospital, Philadelphia, PA 19107, USA; michael.trakhtorchuk@jefferson.edu (M.T.); alessandro.napoli@jefferson.edu (A.N.); 5 Jefferson Moss-Magee Rehabilitation Hospital, Philadelphia, PA 19141, USA; tfg004@jefferson.edu; 6 Physical Medicine and Rehabilitation, Thomas Jefferson University Hospital, Philadelphia, PA 19107, USA; kristin.gustafson@jefferson.edu; 7 Department of Neurological Surgery, Thomas Jefferson University Hospital, Philadelphia, PA 19107, USA; james.harrop@jefferson.edu (J.S.H.); caio.matias@jefferson.edu (C.M.M.); 8 Center for Outcomes and Measurement, Thomas Jefferson University Hospital, Philadelphia, PA 19107, USA; maryjane.mulcahey-hershey@jefferson.edu; 9 Department of Orthopedic Surgery, Rothman Orthopaedics, Philadelphia, PA 19107, USA; alex.vaccaro@rothmanortho.com

**Keywords:** transcutaneous spinal cord stimulation, high-density surface EMG, spinal cord injury, neuromodulation, biomarker, parameter optimization, spatial recruitment, paired-pulse suppression, motor evoked response, dose–response

## Abstract

Transcutaneous spinal cord stimulation (tSCS) is a promising neuromodulation approach for motor recovery after spinal cord injury (SCI), yet clinical programming remains largely dependent on subjective parameter selection. This study evaluated high-density surface EMG (HD-sEMG)–derived spatial and temporal features as objective biomarkers for tSCS optimization in three adults with chronic cervical SCI. A 64-channel electrode array recorded stimulation-evoked responses across five cervical stimulation levels, four pulse widths, and graded amplitudes. Features describing activation magnitude, spatial distribution, cluster morphology, and temporal dynamics were extracted from epoch-based activation maps. Of the three enrolled participants, two demonstrated measurable stimulation-evoked responses and contributed to the paired-pulse analyses, whereas pulse-width analyses were limited to a single responsive muscle (left flexor carpi) in one participant. Paired-pulse analysis identified root mean square (RMS) as the most discriminative feature, revealing nonlinear, muscle- and level-specific dose–response relationships in which maximal suppression often occurred at intermediate rather than maximal amplitudes. Increasing pulse width expanded the spatial extent of recruitment (active area: *p* = 0.006; convex hull area: *p* = 0.004) without altering response timing. Polarity reversal analysis demonstrated stable innervation zone localization across stimulation levels and amplitudes. These findings establish a spatially resolved HD-sEMG framework that may support individualized tSCS parameter selection in SCI.

## 1. Introduction

Spinal cord injury (SCI) disrupts descending motor pathways and often results in severe, lasting impairments in voluntary motor control. Transcutaneous spinal cord stimulation (tSCS) has emerged as a promising non-invasive neuromodulation approach capable of activating spinal circuitries and facilitating motor responses below the level of injury [1,2]. However, the clinical application of tSCS is constrained by the fact that its key stimulation parameters—amplitude, pulse width, frequency, and vertebral level of electrode placement—are selected empirically, with clinicians relying on subjective indicators such as visible movement and therapist observation to tune settings for individual patients [3,4]. Stimulation parameters profoundly influence neural activation and therapeutic outcomes [2], yet no standardized or data-driven framework exists to replace the current trial-and-error approach [5].

The primary electrophysiological tool to assess tSCS-evoked motor responses is conventional surface electromyography (EMG), typically recorded from single bipolar electrode pairs placed over target muscles to quantify outcomes such as peak-to-peak amplitude, root mean square (RMS) values, onset latency, and M-wave characteristics along stimulus–response recruitment curves [1,6]. Use of sEMG in neurorehabilitation after SCI provides a complimentary assessment to detect incomplete lesions: muscle with absent motor scores but present sEMG [7]. While these measures have proven useful for characterizing gross spinal cord excitability—for instance, correlating resting motor threshold and peak-to-peak amplitudes of spinal motor evoked responses with clinical function [8,9]—they carry fundamental limitations. Single-channel or low-density EMG provides only a one-dimensional, spatially averaged representation of muscle activation: it cannot resolve the spatial distribution of motor unit recruitment across the muscle, detect subthreshold or spatially distributed responses that fall below the detection sensitivity of a single electrode pair, or characterize how the spatial organization of muscle activation changes in response to different stimulation parameters [10]. Zheng et al. (2020) demonstrated directly that conventional single-channel sEMG can reveal complex neuromuscular changes in paralyzed muscles following SCI, but that its diagnostic power is substantially inferior to spatially resolved approaches—specifically, high-density surface EMG (HD-EMG) combined with spatial filtering methods yielded significantly greater sensitivity in detecting neuromuscular changes in paralyzed muscles [10]. These findings underscore that the current reliance on spatially impoverished EMG metrics leaves clinicians unable to objectively assess how tSCS parameters reshape the spatial organization of motor output—a critical gap given that the therapeutic goal of tSCS is precisely to modulate the excitability and recruitment patterns of spinal motor pools.

The existing literature lacks any investigation that combines HD-EMG spatial mapping with systematic tSCS parameter tuning to objectively characterize how changes in stimulation amplitude reshape the topographical distribution of muscle activation. Despite its proven ability to resolve motor unit firing properties and spatial activation patterns in SCI [11], HD-sEMG has not been applied to assess or guide tSCS in neuromodulation applications, where motor outcomes continue to be assessed through single-channel amplitude and threshold metrics [8]. This convergence gap means that a fundamental question remains unanswered: how does the spatial organization of motor pool recruitment change as a function of tSCS amplitude, and can these spatial features serve as objective, quantitative biomarkers to guide parameter selection?

The objective of this study was to develop and evaluate high-density EMG (HD-EMG)–derived spatial features as objective biomarkers to guide stimulation parameter optimization during transcutaneous spinal cord stimulation in individuals with spinal cord injury.

## 2. Materials and Methods

Three adults with chronic cervical SCI participated in this study. Inclusion and exclusion criteria are summarized in Table 1. Demographic information for the SCI participants is summarized in Table 2. All participants provided written informed consent prior to enrollment.

### 2.1. Initial Electrophysiological Assessment of Neural Connectivity

The three enrolled participants with SCI first completed a Scanning Session, as described in [12], to assess muscle excitability through peripheral nerve stimulation. Electrical stimulation was delivered using a Chattanooga Continuum portable neuromuscular electrical stimulation (NMES) unit (Enovis™, Lewisville, TX, USA). The purpose of this session was to identify muscles that demonstrated responsiveness to transcutaneous electrical stimulation applied below each participant’s level of motor impairment. Muscle selection was therefore based on the presence of measurable stimulation-evoked activity rather than voluntary motor function which can be present even for individuals who were classified as AIS A based on clinical examination. Only individuals who exhibited at least one responsive muscle were eligible to continue in the study.

Eligible participants then completed two additional study visits during which the neurophysiological effects of transcutaneous spinal cord stimulation were investigated. Specifically, these sessions focused on characterizing reflex modulation mechanisms at different spinal levels and evaluating how stimulation amplitude, pulse width, and waveform type influenced muscle responses.

### 2.2. Electrode Placement and Stimulation Setup

Transcutaneous spinal cord stimulation (tSCS) was delivered using self-adhesive hydrogel electrodes (PALS^®^, Axelgaard, Fallbrook, CA, USA) and the Reynold Innovative Spinal Electrical Stimulation (RISES) system [12].

**Figure 1:**
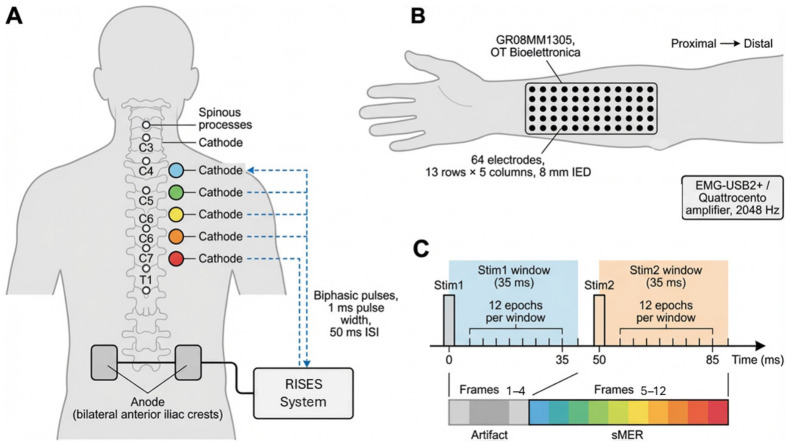
(**A**) Schematic of transcutaneous spinal cord stimulation (tSCS) electrode placement. A circular paravertebral electrode was positioned at cervical intervertebral levels (C3–4, C4–5, C5–6, C6–7, C7–T1) and served as the cathode during the first phase of the biphasic pulse. Two rectangular electrodes placed bilaterally on the anterior iliac crests served as the anodes. Charge-balanced symmetric biphasic pulses (1 ms per phase) were delivered using the RISES stimulation system. (**B**) High-density surface EMG (HD-sEMG) recording configuration. A 64-channel electrode array (GR08MM1305, OT Bioelettronica, Torino, Italy; 5 × 13 grid, 8 mm inter-electrode distance) was placed over the representative target muscle and recorded in monopolar configuration. (**C**) Paired-pulse stimulation paradigm. Two stimuli (Stim1 and Stim2) were delivered with a 50 ms interstimulus interval. EMG responses were analyzed within 35 ms windows following each stimulus and divided into 12 temporal epochs, where the off-line analysis showed that frames 1–4 corresponded primarily to stimulation artifact and frames 5–12 to spinally evoked motor responses.

tSCS was delivered in the form of charge-balanced, symmetric, biphasic rectangular pulse with a duration of 1 ms per pulse (resulting in a total biphasic pulse width of 2 ms). The paravertebral electrode served as the cathode during the first phase and the anode during the second phase of the biphasic pulse. The paravertebral electrode was referred to as the “cathode” and the return electrode as the “anode”. For investigations focused on reflex attenuation at different spinal levels, pairs of stimuli with an interstimulus interval of 50 ms were applied at varying amplitudes for each spinal level and for each participant. The paired-pulse paradigm was employed to assess short-term modulation of spinally evoked motor responses. Comparison of the response to the second stimulus (Stim2) relative to the first stimulus (Stim1) provided a measure of paired-pulse suppression, enabling evaluation of spinal excitability and identification of HD-sEMG features sensitive to stimulation-induced modulation. For pulse width analysis, three pulses were delivered with an interstimulus interval of 3 s for each stimulation intensity as depicted in Figure 1A,B.

The maximum range of the amplitude was individualized and established according to each participant’s maximum comfortable tolerance level.

Electrode placement consisted of a circular electrode positioned at the spinal level of interest, accompanied by two rectangular interconnected electrodes placed bilaterally on the anterior iliac crests.

### 2.3. Data Collection Procedure

High-density surface electromyography HD-sEMG, (grid/electrode model reference: GR08MM1305) was recorded in monopolar (referenced) configuration using a semi-disposable 64-channel electrode array (OT Bioelettronica, Torino, Italy) placed on the skin overlying the target muscle. The array comprised 64 electrodes (diameter: 1 mm) arranged in a 5 × 13 grid (with one absent corner electrode) at an inter-electrode distance of 8 mm. A reference strap was placed on the wrist as depicted in Figure 1B.

Target muscles were selected for each participant based on the residual innervation identified during a preliminary scanning session and included the flexor carpi radialis (FCR), extensor carpi radialis (ECR), biceps brachii (BB), triceps brachii (TB), and first dorsal interosseous (FDI) of the upper extremity. Not all muscles were tested in every participant; muscle selection was individualized according to the neurological level and completeness of injury.

Signals were amplified (gain: 500–2000×), band-pass filtered (100–900 Hz), and digitized at 2048 Hz with 12-bit resolution using a 384-channel EMG amplifier (EMG-USB2+, OT Bioelettronica, Torino, Italy). The HD-sEMG signals were acquired using the default OT Bioelettronica acquisition settings, including a hardware/software band-pass filter of 100–900 Hz implemented within the OTBiolab+ recording system. All analyses were performed on the recorded signals using identical acquisition settings across participants and stimulation conditions. Stimulation timing markers were recorded synchronously with the HD-sEMG data via an auxiliary analog input channel of the amplifier, ensuring precise temporal alignment between stimulation events and evoked muscle responses.

### 2.4. Experimental Protocol

The stimulation protocol comprised three sequential phases designed to systematically identify the optimal stimulation site, pulse width, and waveform for each participant.

#### 2.4.1. Phase 1—Spinal Level Mapping

Paired-pulse stimulation was delivered at five cervical intervertebral levels (C3–4, C4–5, C5–6, C6–7, and C7–8) as depicted in Figure 1A. At each level, stimulation intensity was increased from 5 mA in 5 mA increments until the participant’s maximum tolerance threshold was reached (defined as the highest intensity reported as tolerable without discomfort). HD-sEMG responses were recorded from the target muscles at each intensity to construct level-specific recruitment profiles.

#### 2.4.2. Phase 2—Pulse Width Optimization

The most responsive spinal level—defined as the level eliciting the highest root mean square (RMS) EMG amplitude at the lowest stimulation intensity—was selected for pulse width testing. At this level, four pulse widths were evaluated (200, 400, 800, and 1000 µs). For each pulse width, stimulation intensity was again ramped from 5 mA in 5 mA increments to maximum tolerance. At each intensity step, three biphasic pulses were delivered and the evoked HD-sEMG responses were recorded.

A rest interval of 3 s was provided between successive stimulation levels, pulse widths, and waveform conditions to minimize carryover effects. The order of [levels/pulse widths/waveforms] was fixed from low to high.

### 2.5. Data Analysis

#### 2.5.1. HD-EMG Preprocessing

HD-sEMG signals (2048 Hz, 64 channels) were processed in OTBiolab+ (v1.6.0) and MATLAB (2025a, MathWorks, Natick, MA, USA). Longitudinal single-differential derivation was applied along the proximodistal axis of the electrode grid (i.e., along the muscle fiber direction) to enhance propagating motor unit action potential components and suppress common-mode noise, far-field potentials, and cross-talk [13,14]. This operation computed the difference between adjacent electrodes within each column, reducing the 13 × 5 monopolar grid to a 12 × 5 grid of differential channels mapped to their physical electrode positions (rows: proximodistal; columns: mediolateral). The 64-channel HD-sEMG array consisted of electrodes arranged in a 13 × 5 grid with one absent corner electrode. Following longitudinal single-differential derivation along the proximodistal axis, the processed activation maps were represented as 12 × 5 differential channel grids. One column containing fewer than 12 channels was padded by duplicating the terminal channel to maintain a consistent matrix size.

Stimulation onsets and amplitudes were identified from the auxiliary channel via peak detection. For each paired-pulse stimulus, two 35 ms post-stimulus windows were defined (Stim1 and Stim2). Each window was subdivided into 12 non-overlapping epochs; within each epoch, EMG samples were time-averaged per channel to produce 12 × 5 spatial activation maps. Maps were smoothed with a Gaussian spatial filter (σ = 1 electrode) to reduce spatial noise.

#### 2.5.2. Feature Extraction

From each 12 × 5 epoch map, scalar features were extracted spanning five categories: global amplitude and spatial extent, spatial localization, cluster morphology, distributional statistics, and temporal response characteristics (Table 3).

To distinguish stimulation artifact from neurophysiological spinal motor evoked responses (sEMRs), peak amplitudes were detected across frames using a prominence-based method. Peak timings pooled across all conditions were clustered with a two-component Gaussian mixture model; the midpoint between cluster means served as the data-driven boundary separating artifact (early component) from sEMR (late component).

A stimulation amplitude was classified as suprathreshold if, in the Stim1 condition, the RMS across frames that were classified as sEMR cluster exceeded the baseline RMS (frames with the lowest peak value) by more than threefold. This criterion, analogous to signal-to-noise ratio thresholds commonly applied in evoked potential detection [15,16] and EMG onset analysis [8], ensured that only responses clearly distinguishable from background activity were included in a subsequent analysis.

Stim1–Stim2 comparisons were performed on paired feature values matched by subject, muscle, level, amplitude, and sEMR cluster frames. Paired differences (Stim2 − Stim1) were tested with Wilcoxon signed-rank tests, with paired *t*-tests computed as parametric references. *p*-values were adjusted for multiple comparisons using the Benjamini–Hochberg false discovery rate (FDR). Effect sizes were quantified as Cohen’s $d_{z}$. Features were ranked by a composite discriminability score defined as *Score* prioritizing features with both large effect sizes and strong statistical evidence.

$$d_{z}=\frac{\Delta }{SD(\Delta )}$$

where Δ represents paired differences.

$$Score=\left|d_{z}\right|\times (-{log}_{10}\left(q\right))$$

#### 2.5.3. Comparison Across Stimulation Levels in Paired-Pulse Paradigm

For the most discriminative feature (RMS), percent suppression was calculated for each matched experimental unit (subject × muscle × level × amplitude) using frames sEMR cluster frames (frames 5–12) as:

$$Percent\;Suppression=\;\frac{Stim1-Stim2}{Stim1}\times 100$$

#### 2.5.4. Dose–Response Analysis

To characterize amplitude-dependent modulation, RMS values from Stim1 and Stim2 were averaged across frames 5–12 for each participant × muscle × level × amplitude unit. Positive values indicate suppression; negative values indicate facilitation. Suppression was plotted against stimulation amplitude to identify suppression onset, monotonicity, and the amplitude producing maximal suppression.

#### 2.5.5. Temporal Biomarker Analysis

Three temporal biomarkers were extracted from the RMS waveform within frames 5–12 (each frame corresponds to 3 ms time windows) for each participant × muscle × level unit: (1) *peak frame*—the frame index of maximum RMS; (2) *temporal centroid*—the amplitude-weighted center of mass of the RMS signal; and (3) *early–late ratio*—summed RMS in frames 5–7 divided by frames 10–12. Median values across amplitudes were computed for each combination to yield robust timing estimates.

### 2.6. Pulse-Width Comparison

#### Spatial and Temporal Recruitment Analysis

For pulse width analysis, each trial comprised 10 frames; analyses were restricted to frames 2–10 to exclude pre-response baseline. For each pulse width × amplitude condition, the temporal peak frame was defined as the first frame reaching maximum RMS. Spatial recruitment at peak was quantified by active area (electrode count above threshold) and convex hull area of the suprathreshold region.

Multivariable linear regression (OLS) was used to estimate the independent effects of pulse width and amplitude on three dependent variables: peak frame, active area at peak, and convex hull area at peak. Both predictors were entered as continuous variables. Statistical significance was set at α = 0.05.

### 2.7. Innervation Zone Estimation

Candidate innervation zones (IZ) were identified from polarity reversal patterns in HD-sEMG recorded from the flexor carpi radialis (PS01). For each stimulus, a 36 ms post-stimulus window was extracted and demeaned using a pre-response baseline (0–3 ms). Frames 1–4 (artifact-dominated) were zeroed; analysis was restricted to frames 5–12.

Normalized cross-correlation was computed between adjacent differential channel pairs over a ±2 ms lag range. The channel boundary exhibiting the strongest negative correlation (polarity inversion) was identified as the IZ candidate for each grid column. A single IZ estimate per stimulus was obtained as the modal boundary across columns. Consistency of IZ location was assessed across spinal levels and amplitudes.

## 3. Results

Among the three enrolled participants, Participant 3 did not respond to the transcutaneous electrical stimulation intervention.

**Figure 2:**
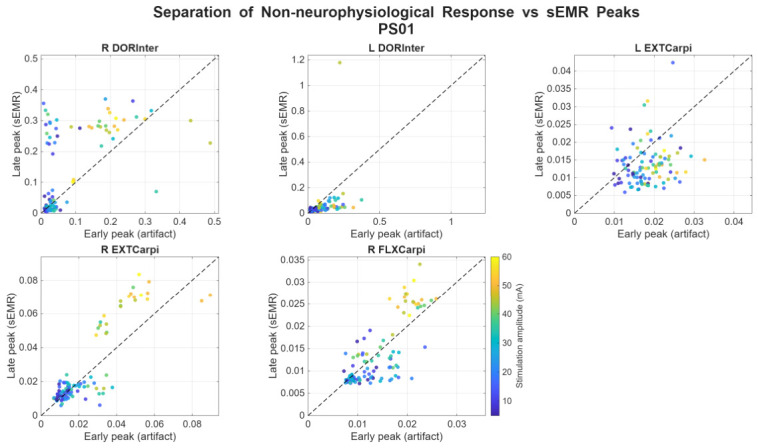
Scatter plots show the relationship between maximum early EMG peak dominated by stimulation artifact (frames 1–4; x-axis) and the maximum late EMG peak corresponding to spinally evoked muscle responses (frames ≥5 y-axis) across muscles in Participant 01 (PS01). Muscles included are the right first dorsal interosseous (R DORInter), left first dorsal interosseous (L DORInter), left extensor carpi radialis (L EXTCarpi), right extensor carpi radialis (R EXTCarpi), and right flexor carpi radialis (R FLXCarpi). Each point represents one stimulation condition (spinal level × amplitude) for Stim1 condition, with color indicating stimulation amplitude (mA). The dashed unity line (y = x) denotes equal early and late peak amplitudes, points above this line indicate late responses exceeding early non-neurophysiological response, consistent with spinally evoked activation. The figure demonstrates muscle-specific and amplitude-dependent recruitment patterns, supporting the separation of neurophysiological responses from stimulation artifact.

Consistent with the Gaussian mixture modeling approach described in the Methods, two distinct peak components were identified across frames. The early component peaked around frame 3 and corresponded to stimulation artifact, whereas the late component peaked around frame 8 and corresponded to sEMRs. The midpoint between these clusters means defined a separation boundary at frame 5, which was subsequently used to distinguish artifact-dominated responses from neurophysiological sEMGs in the analysis as depicted schematically in Figure 1. Figure 2 depicts distinction of stimulation artifact from neurophysiological responses for a representative participant (PS01). The maximum early EMG peak (frames 1–4), dominated by stimulation artifact, was plotted against the maximum late EMG peak (frames ≥5), corresponding to spinally evoked muscle responses for each stimulation condition. Muscles included are the right first dorsal interosseous (R DORInter), left first dorsal interosseous (L DORInter), left extensor carpi radialis (L EXTCarpi), right extensor carpi radialis (R EXTCarpi), and right flexor carpi radialis (R FLXCarpi). Conditions falling above the unity line (x = y) indicate late responses exceeding the early artifact-dominated peak, consistent with the presence of spinally evoked activation. All subsequent analyses were restricted to frames 5–12.

**Figure 3:**
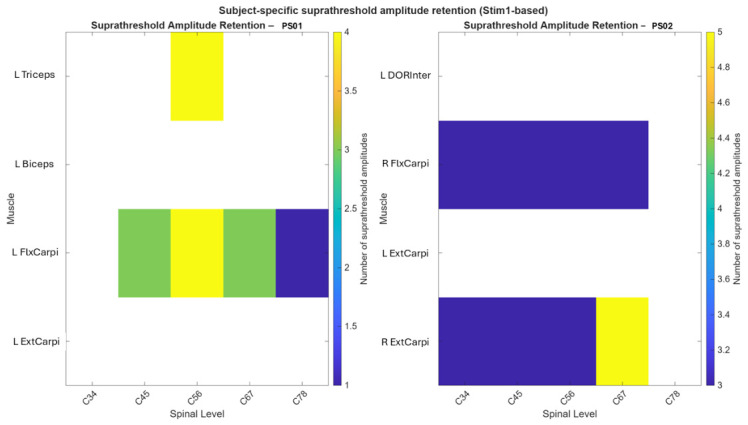
Heatmaps display the number of unique suprathreshold stimulation amplitudes retained for each muscle (y-axis) at each spinal level (x-axis) in PS01 and PS02. Color intensity represents the count of retained suprathreshold amplitudes, with warmer colors indicating greater retention. Blank cells indicate no suprathreshold responses detected for the corresponding muscle–level combination. These subject-specific patterns illustrate differential muscle recruitment and variability in responsiveness across spinal levels.

Applying the suprathreshold criterion resulted in the retention of stimulation amplitudes that produced RMS values during the sEMR frames exceeding baseline RMS by more than threefold. The number of retained suprathreshold amplitudes varied across muscles and spinal stimulation levels: PS01 exhibited broad suprathreshold activation across multiple muscles and spinal levels, whereas PS03 showed more selective patterns with higher activation thresholds and fewer responsive muscles, reflecting differences in recruitment thresholds and response sensitivity. The distribution of retained suprathreshold amplitudes for each muscle and spinal level is shown in Figure 3.

### 3.1. Paired-Pulse Analysis

#### 3.1.1. Identification of the Most Discriminative Features

**Figure 4:**
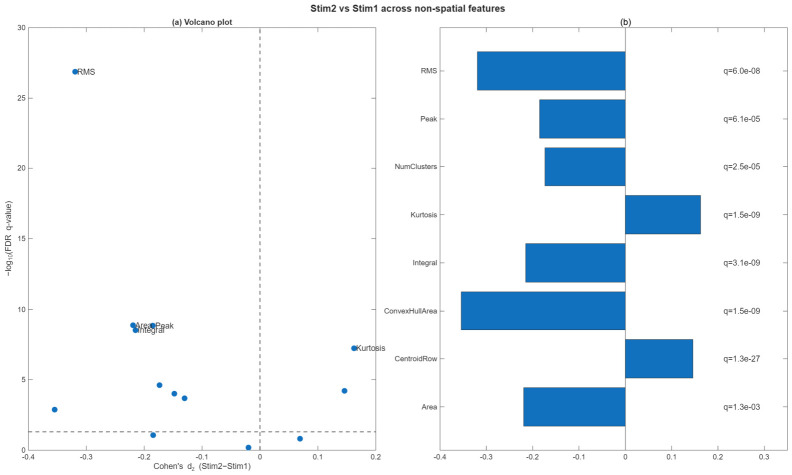
(**a**) Volcano plot showing effect size (Stim2 − Stim1) versus FDR-adjusted significance for each feature for frames 5–12. Negative values indicate lower feature values during Stim2 relative to Stim1. The dashed horizontal line marks the FDR significance threshold (q = 0.05). Magnitude-based features, including RMS, Area, Integral, and Peak, show consistent reductions during Stim2 with strong statistical support. (**b**) Ranked effect sizes for the most discriminative features. Bars represent the magnitude and direction of the condition difference, and corresponding FDR-adjusted q-values are displayed. Overall, differences between conditions are primarily driven by changes in activation magnitude rather than distributional or shape-related metrics.

Feature-level comparisons between Stim1 and Stim2 responses are shown in Figure 3. The volcano plot (Figure 4a) indicates that several features exhibited significant negative effect sizes, reflecting reduced responses during Stim2 relative to Stim1. Notably, the features showing the strongest reductions were predominantly magnitude-related metrics, including RMS, peak amplitude, signal integral, and suprathreshold area. The feature ranking (Figure 4b) further confirms RMS as the most discriminative feature between conditions. Spatial descriptors, such as convex hull area and centroid position, showed smaller but significant reductions, whereas distribution-based features (e.g., kurtosis and entropy) showed smaller or positive effect sizes.

#### 3.1.2. Level-Specific Suppression

**Figure 5:**
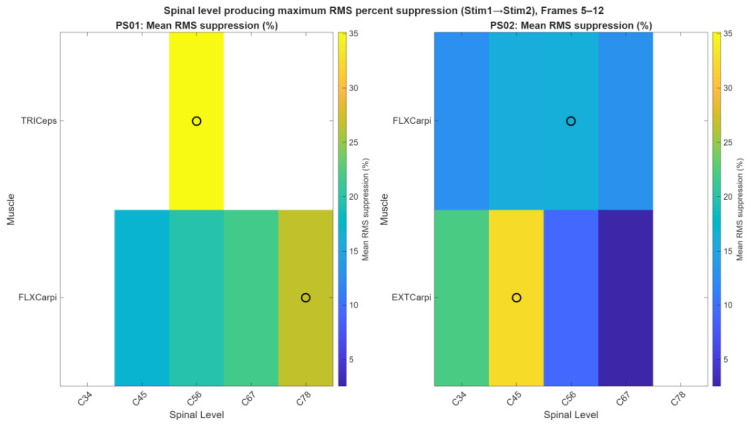
Spinal level producing maximum RMS percent suppression (Stim1 → Stim2) across Frames 5–12. Heatmaps show mean RMS suppression (%) for each muscle–level combination in PS01 (**left**) and PS02 (**right**). Color intensity represents the magnitude of suppression, with warmer colors indicating greater percent reduction in RMS following Stim2. Black circles denote the spinal level yielding the maximum suppression for each muscle within each subject. Suppression patterns demonstrate subject- and muscle-specific level selectivity.

Level- and muscle-dependent suppression patterns are illustrated in Figure 5. The heatmaps display the mean RMS percent suppression across spinal stimulation levels (C34–C78), with warmer colors indicating greater reductions in RMS following Stim2. In PS01 (left panel), maximal suppression occurred at C78 for L FLXCarpi (26.7%) and at C56 for L TRICeps (35.1%), highlighted by black circles marking the level of maximum suppression. In PS02 (right panel), the strongest suppression was observed at C45 for R EXTCarpi (32.6%) and C56 for R FLXCarpi (16.9%). These patterns demonstrate that the spinal level producing maximal suppression differs across muscles and participants, indicating subject- and muscle-specific segmental selectivity in paired-stimulation responses.

#### 3.1.3. Amplitude-Dependent Recruitment Dynamics

In PS01, several muscle–level combinations exhibited inverted-U dose–response profiles, with maximal suppression occurring at intermediate stimulation amplitudes. For example, triceps at C5–6 peaked at approximately 35 mA (~51% suppression), while FCR at C4–5 peaked at 30 mA (~49%) before reversing toward facilitation at higher amplitudes. Other spinal levels showed distinct patterns: C6–7 alternated between facilitation and suppression across amplitudes, whereas C7–8 demonstrated suppression only at higher stimulation intensities.

**Figure 6:**
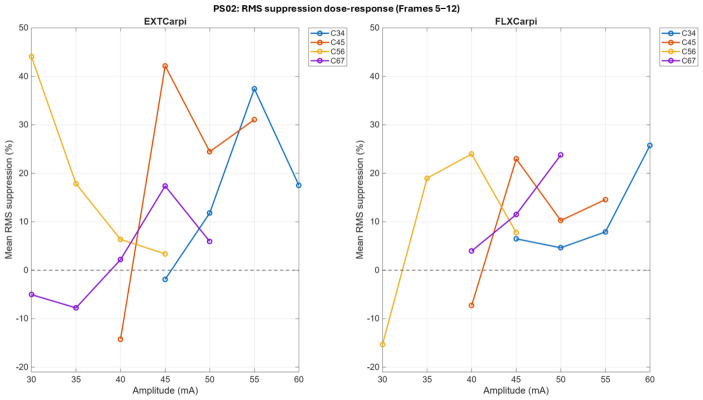
Dose–response relationship of RMS percent suppression (Stim1 → Stim2) across stimulation amplitudes (Frames 5–12) in a representative SCI participant (PS02). (**Left panel**) EXTCarpi5; (**Right panel**) FLXCarpi3. Colored traces indicate spinal levels (C34, C45, C56, C67). Positive values denote reduced RMS after Stim2 relative to Stim1, whereas negative values indicate facilitation. Suppression magnitude varies across both amplitude and spinal level, demonstrating non-uniform amplitude dependence and muscle-specific segmental effects within the same participant.

In PS02 (Figure 6), dose–response heterogeneity was similarly prominent. ECR at C5–6 showed maximal suppression at the lowest tested amplitude (30 mA, ~44%) with progressive attenuation, whereas C4–5 peaked at 45 mA (~42%). C3–4 showed near-monotonic suppression increases with amplitude; C6–7 exhibited bidirectional transitions. FCR responses varied by level, with intermediate amplitudes producing peak suppression at some levels (e.g., C5–6) and gradual increases at others.

Across all muscle–level combinations, maximal suppression occurred more frequently at intermediate amplitudes than at the highest intensity tested, suggesting a non-monotonic dose–response relationship in which suprathreshold increases in stimulation intensity do not uniformly enhance post-activation depression.

#### 3.1.4. Temporal Biomarker Analysis

**Figure 7:**
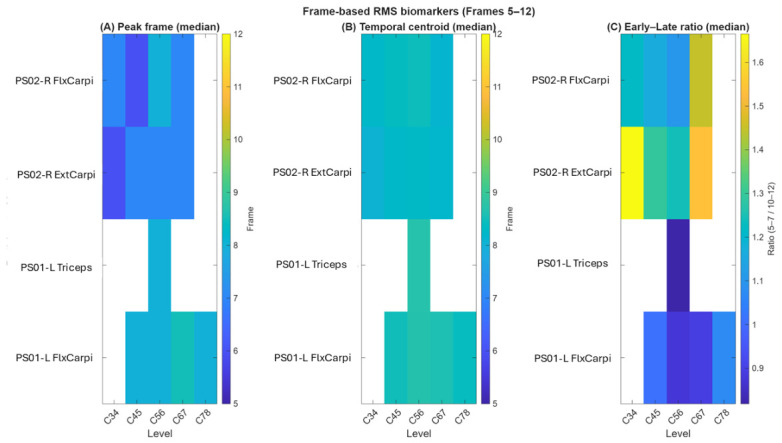
Frame-based RMS biomarkers illustrating amplitude- and spinal-level–invariant localization of the innervation zone across muscles and participants. Heatmaps display median values across retained suprathreshold amplitudes for frames 5–12. (**A**) Median peak frame indicating the time frame at which maximal RMS activity occurs. (**B**) Median temporal centroid representing the center of mass of RMS activity across frames. (**C**) Median early-to-late ratio (frames 5–7 relative to frames 10–12) quantifying the temporal distribution of activation within the response window. Rows correspond to participant–muscle combinations and columns represent spinal stimulation levels (C34–C78). Consistent temporal localization across levels suggests that the timing characteristics of the evoked response remain largely stable despite changes in stimulation amplitude and spinal level, supporting the presence of a robust and invariant innervation zone signature.

Temporal characteristics of the evoked responses are summarized in Figure 7. Across participants and muscles, median peak frames were confined to a narrow temporal window (frames 7–9). In PS01, L FLXCarpi exhibited peak frames between 8 and 9 across spinal levels, while L TRICeps showed peak activity consistently around frame 8. In PS02, both R FLXCarpi and R EXTCarpi demonstrated peak frames primarily between 7 and 8, indicating minimal variation in response latency across stimulation levels.

Temporal centroid values closely paralleled the peak frame distributions, remaining centered near frames 8–9 across muscles and levels (Figure 7B), further supporting stable temporal localization of the response. In contrast, the early–late ratio revealed muscle-dependent differences in the temporal distribution of activation. In PS02, R EXTCarpi exhibited higher early-weighted responses (ratio > 1.4 at several levels), whereas PS01 muscles showed ratios closer to unity, indicating a more balanced early–late distribution (Figure 7C).

A one-way Kruskal–Wallis test demonstrated significant effects of muscle (*p* = 2.24 × 10^−11^) and spinal level (*p* = 0.000133) on peak frame timing in PS01.

### 3.2. Pulse-Width Effects: Spatial and Temporal Recruitment Analysis

**Figure 8:**
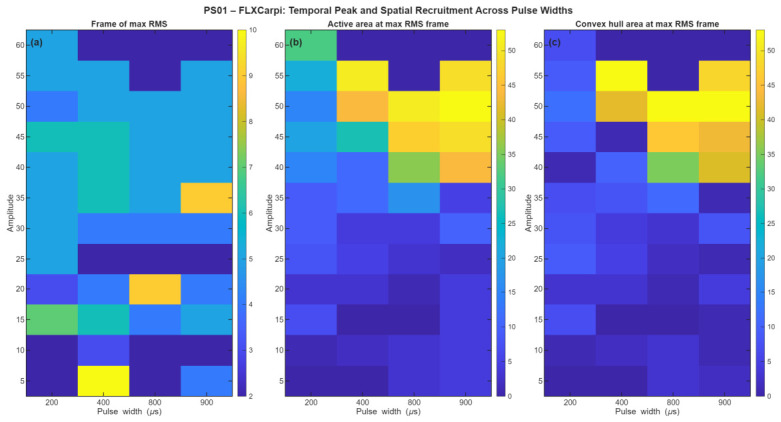
(**a**) Frame of maximal RMS (frames ≥ 2) across pulse widths and stimulation amplitudes. Increasing pulse width did not systematically shift the timing of peak activation. (**b**) Active area at the frame of maximal RMS. (**c**) Convex hull area at the frame of maximal RMS. In contrast to peak timing, both spatial metrics increased with pulse width at comparable amplitudes, indicating broader spatial recruitment with longer pulse durations. Heatmaps display amplitude (rows) and pulse width (columns).

Data for this analysis was available only for PS01 and L FlxCarpi muscle. The results of the linear model controlling for amplitude showed no association between pulse width and peak timing (β = −2.8 × 10^−4^ frames/µs, 95% CI [−2.22 × 10^−3^, 1.66 × 10^−3^], *p* = 0.772) as depicted in Figure 8a. In contrast, pulse width was positively associated with spatial recruitment metrics evaluated at the peak RMS frame as depicted in Figure 8b,c. After adjusting for amplitude, active area increased with pulse width (β = 0.01499 units/µs, 95% CI [0.00450, 0.02547], *p* = 0.006; R^2^ = 0.70), and convex hull area also increased with pulse width (β = 0.01943 units/µs, 95% CI [0.00643, 0.03243], *p* = 0.004; R^2^ = 0.53), indicating broader spatial recruitment at longer pulse widths.

### 3.3. Innervation Zone Estimation

Polarity reversal analysis revealed a consistent clustering of inversion boundaries around the mid-grid region across stimulation amplitudes and spinal levels. For each stimulation condition, the row boundary corresponding to the strongest polarity reversal was extracted for each grid column. Boundary indices were then pooled across amplitudes within each spinal level and summarized using mean ± standard deviation (SD).

The mean polarity reversal boundary index across amplitudes was 6.94 ± 0.77 (mean ± SD) for C4–C5 level, 7.09 ± 0.78 for C5–C6 level, 6.67 ± 1.03 for C6–C7 level, and not detectable for C7–C8 level due to smaller response magnitude.

When pooled across all amplitudes and both spinal levels, the overall mean polarity reversal boundary index was 7.03 ± 0.78.

## 4. Discussion

The primary contribution of this work is methodological rather than mechanistic. The present analyses were conducted exclusively in individuals with spinal cord injury (SCI), a population characterized by substantial heterogeneity in lesion level, chronicity, residual descending pathways, and muscle availability. In the absence of a healthy control cohort, the current data do not allow conclusions regarding how transcutaneous spinal cord stimulation (tSCS) modulates intact neural pathways or whether the observed temporal and amplitude-dependent patterns reflect specific spinal circuit mechanisms.

Instead, the strength of this study lies in demonstrating a subject-specific, frame-based analytical framework using high-density EMG. By quantifying peak frame, temporal centroid, early–late energy distribution, and percent suppression within defined post-stimulation windows, we provide a structured approach for evaluating stimulation effects with higher temporal precision than amplitude-averaged metrics alone. This framework enables direct comparison across muscles, spinal levels, and participants while accommodating inter-individual variability.

Interpretation of these findings should consider the analysis-specific sample sizes. Although three individuals with SCI were enrolled, one participant did not demonstrate measurable stimulation-evoked responses and therefore did not contribute to the paired-pulse analyses. Consequently, the paired-pulse findings are based on two participants. Participant PS03, who sustained a non-traumatic spinal cord injury secondary to a benign spinal tumor, did not exhibit measurable stimulation-evoked responses. The underlying mechanism for this lack of responsiveness for PS03 cannot be determined from the present study but may reflect differences in lesion pathology, preserved circuitry, or peripheral neuromuscular integrity relative to the other participants. Furthermore, pulse-width analyses were available only for the left flexor carpi muscle of a single participant and should be regarded as exploratory. These results primarily establish methodological feasibility and motivate validation in larger cohorts.

Given the marked heterogeneity inherent to SCI, such feature-based analyses may be particularly valuable for personalized neuromodulation strategies, where the goal is not to infer generalized physiology but to identify stimulation parameters that produce meaningful, individual-specific responses.

### 4.1. Comparison Between Stim1 and Stim2 Conditions for Paired-Pulse Analysis

#### 4.1.1. Spinal Level Specificity of Suppression

In this SCI cohort, the spinal level producing maximal RMS attenuation after Stim2 was muscle-specific and varied between participants. This segmental variability likely reflects differences in injury level, spared circuitry, and available motor pools across individuals. These findings highlight the importance of subject-specific optimization of stimulation parameters rather than relying on uniform segmental targets, supporting a personalized approach to neuromodulation in SCI.

#### 4.1.2. Amplitude-Dependent Recruitment Dynamics

The observed amplitude-dependent suppression profiles demonstrate that stimulation effects are not uniformly graded with increasing intensity. Instead, suppression magnitude varied in a nonlinear and spinal level–specific manner, with several muscle–level combinations exhibiting maximal suppression at intermediate amplitudes rather than at the highest intensity tested. In some cases, suppression emerged only beyond a threshold amplitude, while in others, low amplitudes produced facilitation before transitioning to suppression.

These patterns suggest that increasing stimulation intensity does not simply scale neural output but may engage distinct afferent populations and interneuronal circuits at different amplitudes. The presence of inverted-U and bidirectional responses supports the idea that spinal recruitment dynamics are governed by selective circuit activation rather than monotonic motor unit recruitment alone. Such level-specific amplitude optima highlight the importance of individualized parameter selection when designing neuromodulatory interventions.

### 4.2. Comparison Between Different Pulse Widths

This analysis investigated whether pulse width modulates temporal and spatial characteristics of sEMR recruitment in PS02 (FLXCarpi). Increasing pulse width did not systematically shift the frame of maximal RMS, indicating no meaningful change in peak activation timing within frames 2–10. In contrast, pulse width was significantly associated with greater active area and convex hull area at the peak RMS frame, even after adjusting for stimulation amplitude.

These findings suggest that pulse width primarily scales the spatial extent of motor unit recruitment rather than altering temporal activation dynamics. Consistent with strength–duration principles, longer pulse widths likely increase delivered charge and recruit a broader motor unit population without affecting response latency. This dissociation between stable timing and expanded spatial recruitment highlights pulse width as a parameter for modulating recruitment magnitude and distribution while preserving temporal characteristics of the evoked response.

### 4.3. Amplitude and Spinal-Level Invariant Localization of the Innervation Zone

Polarity reversal analysis demonstrated that the estimated innervation zone remained consistently localized near the mid-grid region across stimulation amplitudes and spinal levels. The polarity reversal boundary clustered around row ~7 with minimal variability, indicating that the spatial origin of the evoked muscle activity was stable across stimulation conditions. This stability suggests that the observed responses primarily reflect activation of motor units within a fixed anatomical endplate region rather than shifts in the site of excitation. These findings are consistent with the temporal biomarker analysis (Figure 7B), which showed that the timing of peak RMS and the temporal centroid were confined to a narrow frame window across stimulation levels, indicating stable response latency. In contrast, spatial recruitment metrics (Figure 8) demonstrated that increasing stimulation intensity or pulse width expanded the spatial extent of activation without altering the temporal location of the peak response. Together, these results suggest that while stimulation parameters modulate the magnitude and spatial spread of recruited motor units, the temporal characteristics and anatomical origin of the evoked response remain largely invariant. This dissociation between stable temporal and spatial localization versus parameter-dependent recruitment magnitude supports the use of temporal and polarity-based biomarkers as robust indicators of underlying neuromuscular activation patterns in stimulation-evoked HD-EMG recordings.

### 4.4. Limitations and Future Steps

While the small sample size limits generalizability, the within-subject consistency of the observed patterns supports the feasibility of this approach as a quantitative tool for stimulation parameter optimization. Future work should validate these biomarkers in larger cohorts, assess their test–retest reliability across sessions, and evaluate whether HD-sEMG-guided parameter selection translates to improved functional outcomes during tSCS-augmented rehabilitation.

## 5. Conclusions

This study presents a subject-specific, frame-based analytical framework using high-density surface EMG to characterize motor responses evoked by transcutaneous spinal cord stimulation (tSCS) in individuals with spinal cord injury. Rather than focusing on generalized mechanistic interpretations, the framework emphasizes precise temporal and spatial feature extraction to capture stimulation effects within and across individuals.

Across analyses, stimulation parameters differentially influenced response characteristics. Amplitude and pulse width modulated the magnitude and spatial extent of motor unit recruitment, reflected in RMS amplitude, activation area, and convex hull measures, while temporal features such as peak frame and temporal centroid, as well as innervation zone localization, remained largely stable. This dissociation suggests that spatial features serve as sensitive indicators of stimulation dose, whereas temporal features may reflect intrinsic and preserved neuromuscular properties.

Paired-pulse analyses further revealed that suppression of the second response is highly subject- and spinal level-specific, with nonlinear amplitude-dependent profiles. Notably, maximal suppression frequently occurred at intermediate stimulation intensities, indicating that increasing stimulation does not monotonically enhance neuromodulatory effects but instead engages distinct neural circuits depending on dose and segment.

Taken together, these findings support the utility of feature-based HD-EMG analyses for identifying individualized stimulation signatures in heterogeneous SCI populations. This approach provides a quantitative foundation for optimizing stimulation parameters at the subject level, advancing the development of personalized neuromodulation strategies. Future work should extend this framework to larger cohorts and evaluate its reliability and functional relevance in guiding rehabilitation interventions.

## Figures and Tables

**Table 1:** Inclusion and exclusion criteria for SCI participants.

Inclusion criteria Age 18 years or older. Has a non-progressive or traumatic spinal cord injury. American Spinal Injury Association (ASIA) Impairment Scale (AIS) classification A, B, C, or D. Can participate in physical and occupational therapy rehabilitation programs. Is at minimum 12 months post-injury. Can provide informed consent, as evidenced by the teach-back method. Has adequate care partner support to facilitate participation in study. Exclusion criteria Has uncontrolled cardiopulmonary disease or cardiac symptoms (as determined by investigators). Has any unstable or significant medical condition that is likely to interfere with study procedures or likely to confound study endpoint evaluations such as uncontrolled cardiopulmonary disease or cardiac symptoms, unmanaged neuropathic pain, unmanaged autonomic dysreflexia, unmanaged orthostatic hypotension, or uncontrolled spasticity. Requires ventilator support. Has an autoimmune etiology of spinal cord dysfunction/injury. Has pressure injury in area(s) that will come into contact with electrodes or that has potential to worsen with study participation. Has an implanted medical device (e.g., cochlear implant, pacemaker, neurostimulator, or a powered medication infusion device such as a baclofen pump). Is pregnant, planning to become pregnant, or currently breastfeeding. Has concurrent participation in another drug or device trial that may interfere with this study. Has other traumatic injuries such as peripheral nerve injuries, or severe musculoskeletal injuries that prevent evaluation of response to, or participation in, rehabilitation.

**Table 2:** Demographic information of spinal cord injury participants.

Subject ID	Age	Sex	Cause of Injury	AIS and NLI at Study Baseline	Years Since Injury (Years)
PS01	64	M	Traumatic fall	B sensory incomplete/C6	1
PS02	50	F	Traumatic Sports/Leisure	A motor and sensory complete/C3	10
PS 03	51	M	Non-traumatic tumor—benign	Unknown/C4	10

**Table 3:** Summary of HD-EMG features extracted from epoch-based activation map.

Feature Category	Feature Name	Description	Physiological Interpretation
Global Amplitude	Root Mean Square (RMS)	RMS of all grid elements within an epoch map	Overall magnitude of muscle activation
	Peak Amplitude	Maximum rectified EMG amplitude across all electrodes	Strongest localized muscle activation
	Activation Area	Number of electrodes with |EMG| > 30 µV	Spatial extent of active muscle regions
Spatial Localization and Complexity	Center of Activity (Centroid-Row, Column)	Activity-weighted center of gravity of the activation map	Spatial location of dominant muscle activation
Cluster Morphology	Clusters Count	Connected-component count in suprathreshold binary map	Fragmentation vs. coherence of activation
Spatial Distribution Statistics	Skewness	Skewness of vectorized activation map	Asymmetry of spatial activation distribution
	Kurtosis	Kurtosis of vectorized activation map	Degree of spatial concentration or focality
Spatial Profiles	Row Activation Count	Number of suprathreshold electrodes per row	Longitudinal organization of muscle activation
	Column Activation Count	Number of suprathreshold electrodes per column	Localization of activation hot spots
Temporal Shape Features	Response Duration	Time (ms) EMG remains above threshold after stimulation	Narrow vs. wide sEMR response characterization

## Data Availability

Data are available upon request.
